# Raman Microscopic Identification of Microorganisms on Metal Surfaces via Support Vector Machines

**DOI:** 10.3390/microorganisms10030556

**Published:** 2022-03-03

**Authors:** Thomas J. Tewes, Mario Kerst, Frank Platte, Dirk P. Bockmühl

**Affiliations:** Faculty of Life Sciences, Rhine-Waal University of Applied Sciences, Marie-Curie-Straße 1, 47533 Kleve, Germany; thomasjohann.tewes@hsrw.eu (T.J.T.); mario.kerst@hsrw.org (M.K.); frank.platte@hsrw.eu (F.P.)

**Keywords:** Raman, microorganisms, silver, stainless steel, support vector machine (SVM)

## Abstract

An easy, inexpensive, and rapid method to identify microorganisms is in great demand in various areas such as medical diagnostics or in the food industry. In our study, we show the development of several predictive models based on Raman spectroscopy combined with support vector machines (SVM) for 21 species of microorganisms. The microorganisms, grown under standardized conditions, were placed on a silver mirror slide to record the data for model development. Additional data was obtained from microorganisms on a polished stainless-steel slide in order to validate the models in general and to assess possible negative influences of the material change on the predictions. The theoretical prediction accuracies for the most accurate models, based on a five-fold cross-validation, are 98.4%. For practical validation, new spectra (from stainless-steel surfaces) have been used, which were not included in the calibration data set. The overall prediction accuracy in practice was about 80% and the inaccurate predictions were only due to a few species. The development of a database provides the basis for further investigations such as the application and extension to single-cell analytics and for the characterization of biofilms.

## 1. Introduction

Besides common microbiological identification methods using a combination of cultivation on different nutrition media and additional tests such as Gram staining, there are more powerful tools such as DNA-based methods or matrix-assisted laser desorption ionization-time of flight mass spectroscopy (MALDI-TOF MS). Novel, culture-free methods such as single-cell sequencing [1,2] still have the disadvantage of requiring specific labels. Optical spectroscopy in combination with chemometric methods has allowed the identification of microorganisms as well, and several publications have already shown that reliable identification of bacteria is feasible using Raman spectroscopy [3,4,5]. A quick and easy identification of microorganisms is desirable for a variety of reasons. For example, in medical diagnostics, hours could decide about the health status of a patient, and in food, processing time is crucial to ensure product safety and a long shelf life. In 2002, Maquelin et al. investigated the potential use of vibrational spectroscopies in medical microbiology emphasizing the great potential for clinical diagnostic microbiology assuming a large database of well-defined strains [6]. Ho et al. recently showed that they could distinguish between methicillin-resistant and -susceptible isolates of *Staphylococcus aureus* with 89% accuracy via Raman spectroscopy and deep learning [7]. The results were validated on clinical isolates from 50 patients. By using 10 bacterial spectra from each patient isolate, they achieved treatment identification accuracies of over 99% [7]. Yang and Irudayaraj have shown that FT-Raman spectroscopy can be an excellent tool for rapid screening food surfaces for potential contamination with microorganisms and their classification [8]. More food-related research has been conducted, *inter alia*, to detect the bacterial genus *Brucella*. The results indicate that micro-Raman spectroscopy in combination with support vector machines (SVM) could be a promising alternative for the identification of *Brucella* spp. both on agar plates and directly in milk. Identification at the single-cell level can be achieved within two hours without the need for pre-cultivation [9]. Raman spectroscopy allows for identifying a small amount of biological material and even single cells [10,11], which might allow for skipping time-consuming pre-enrichment and cultivation.

A limitation of Raman spectroscopy in the analysis of microbiological samples, besides fluorescence as competing light, is the relatively weak Raman scattered signal. However, the intensity of the Raman signal can be increased by the substrate used. Mikoliunaite et al. have investigated whether and how the signal enhancement changes depending on the substrate used [12]. Since confocal Raman microscopy aims for investigating surfaces, the height of the sample layer on the substrate material obviously plays a role as well. Very thin sample layers or single cells can allow excellent signals due to reflections and surface enhancement Raman effects, whereas for thicker sample layers the effect of the surface material becomes increasingly less important. In our study, we show the development of several predictive models for 21 microbial species. In a previous publication on Raman microscopic differentiation of conidia, we have already shown that SVMs can achieve very accurate predictions, even among closely related species [13]. For this reason, we have also focused on SVMs in this work. The spectra for model development were obtained from microorganisms placed on a protected silver mirror slide. Additional data were recorded on a different substrate, i.e., polished stainless steel, which is (i) cheaper and (ii) less sensitive to chemical and mechanical stress. Predictions were made for the Raman spectra obtained from the stainless-steel slides in order to validate the models in general and to assess the possible negative influences of the different materials on the predictions. The development of a database will be the basis for further investigations such as the application and extension to single cells and in the characterization of microbial biofilms.

## 2. Materials and Methods

### 2.1. Growth Conditions and Sample Preparation

Glycerol stocks of bacterial and yeast strains were stored at −80 °C. Aliquots were thawed, distributed on universal tryptic soy agar (TSA) plates (Merck, Darmstadt, Germany) or malt extract agar (MEA) (Merck, Darmstadt, Germany) using sterile inoculating loops and incubated at 30 °C for 24 h. A subculture of these cultures was obtained by transfer to other TSA plates and incubated for 24 h. These cultures were used for the investigation. The 21 different microorganisms used and the exact growth parameters, including variations, are listed in Table 1.

One milliliter of 0.9% sterile NaCl solution was pipetted in 1.5 mL reaction tubes. Cell material was collected with a sterile inoculation loop and transferred into the NaCl solution. After thorough mixing with a vortex mixer, the suspension was centrifuged (Heraeus Fresco 17, Thermo Scientific, Dreieich, Germany) for 3 min at 5000× *g* rpm. The supernatant was discarded and the cell pellet with some residual moisture was homogenized with a sterile pipette tip and then 1 µL was transferred to the SiO_2_ protected silver mirror slide (PFR14-P02, Thorlabs, Bergkirchen, Germany) or the highly polished stainless-steel slide (Renishaw, Pliezhausen, Germany), followed by drying for 15 min at 20 °C.

### 2.2. Spectral Recording

To collect Raman spectra, a confocal Raman microscope (inVia, Renishaw, Gloucestershire, UK) was used with an excitation wavelength of 633 nm (helium-neon (HeNe)) and a 100× magnification lens. The detected spectral region was between 606 cm^−1^ to 1736 cm^−1^ with an average resolution of about 1.1 cm^−1^. The spectra were recorded with the 100× lens in a spiral shape from the inside to the outside with a distance of about 2 to 4 µm between the measuring spots. This was carried out to bleach out possible fluorescence interferences. Such spirals were recorded at several locations on the surface of the samples, each comprising 50 to 300 spectra. All spectra have been recorded with an excitation wavelength of 633 nm at about 3.5 mW laser power on the sample and a laser diameter of about 8 µm. The database includes a total of 17651 Raman spectra from 21 species, i.e., an average of about 840 spectra per species. Information about all species in the database, DSM numbers, and additional information about used exposure time, accumulations per spectrum, and growth conditions can be found in Table 1. Please note that the selection of microorganisms in our work intentionally did not focus on pathogenicity or, for example, Gram behavior. The initial aim was to collect data as broadly as possible with microorganisms of which both high Raman spectroscopic similarities and large differences were suspected. For all species that visibly contained color pigments, only 15 accumulations were used, and the spectra were not recorded in a spiral pattern but side by side with a spacing of 4 µm. On the one hand, this procedure should lead to the carotenoids not photodegrading, but on the other hand, the measuring points should still be close enough to each other for a bleaching effect to occur. With this method, the acquisition time for a spectrum is about 30 s (most microorganisms) or 22.5 s (pigmented microorganisms).

### 2.3. Data Preprocessing

For data preprocessing and model development, MATLAB R2021b and the MATLAB Classification Learner R2021b were used (MathWorks, Natick, MA, USA). After the spectra were interpolated, baseline correction and smoothing via low pass filter (LPF) took place. The appropriate LPF code for this can be requested from the authors. All spectra were normalized (z-score) and principal component analysis (PCA) was performed.

The models are based on SVM with a cubic kernel function using different numbers of the first principal components (PCs). The parameters of the SVM were not tuned, but the default settings were used (box constraint level = 1, kernel scale mode: auto, multiclass method one-vs-one, standardize data enabled, hyperparameter options disabled).

## 3. Results and Discussion

### 3.1. Comparison of Different Substrates on the Effects of Raman Spectra of Bacteria

**Figure 1:**
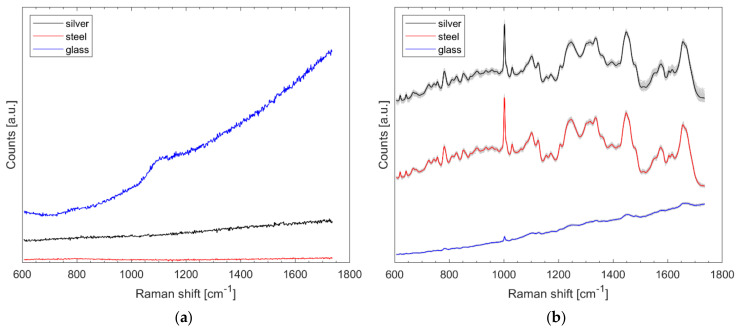
Untreated Raman spectra collected from protected silver mirror slide, stainless-steel slide, and glass slide (**a**) and 85 normalized Raman spectra of *B. diminuta* on each named substrate with arbitrary offset for better visualization (**b**).

Spectra were recorded from the aforementioned silver slide, stainless-steel slide, and from a microscopic glass slide (Gerhard Menzel GmbH, Braunschweig, Germany) with 1.5 s exposure time at about 3.5 mW laser power and 15 accumulations per spectrum. The results of Figure 1a show that all materials do not cause distinct Raman bands, but the glass has a higher total intensity. The spectra of Figure 1a were neither treated nor normalized.

Eighty-five Raman spectra were recorded from the outer edge of each of three *B. diminuta* (*Bdi*) samples (on silver, stainless steel, and glass) using the method described. Care was taken to ensure that the locations where the spectra were recorded were at the same position as the dried bacterial droplet in each case. It is clear from Figure 1b that glass results in the least pronounced signals. The difference between stainless steel and silver is hardly noticeable on average, but the data scatter somewhat more with the protected silver mirror slide (noticeable from the gray spectra in the background) in Figure 1b. For the glass slide, the typical bacterial signatures are only faintly visible without data pretreatment. The spectra of Figure 1b were normalized and presented with an offset; in fact, the total intensity hardly differs here (data not shown).

### 3.2. Pretreated Raman Spectra of Microorganisms on Silver Mirror Slide

**Figure 2:**
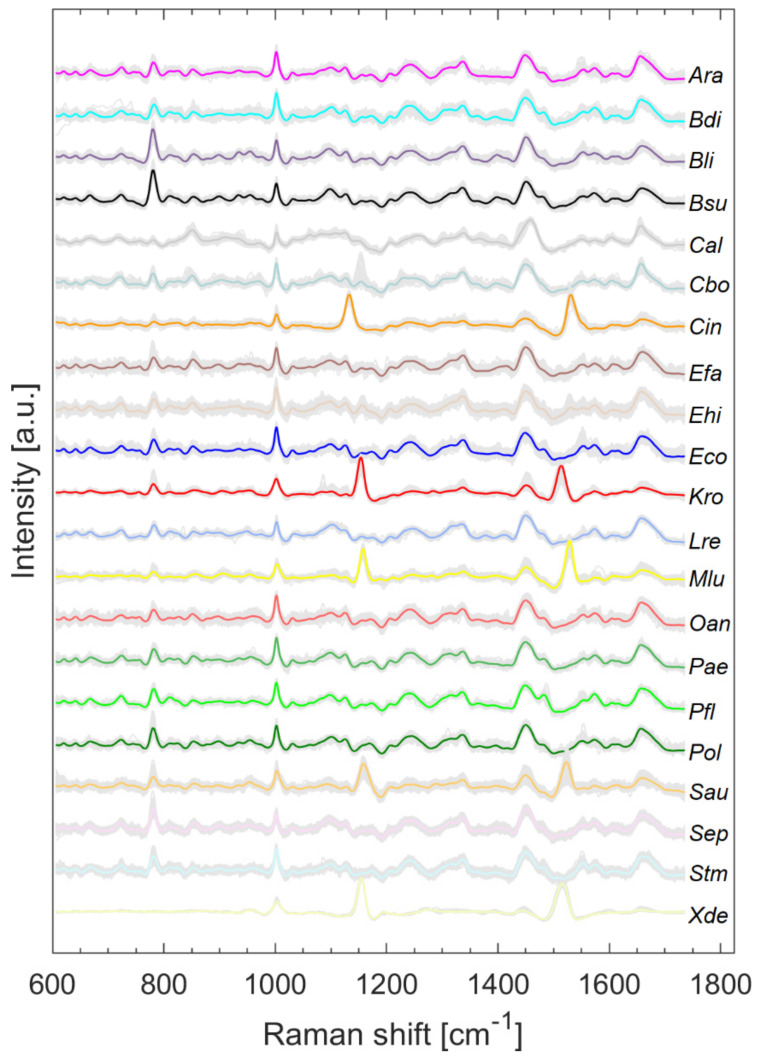
Raman spectra collected from microorganisms on the silver mirror slide after baseline correction, smoothing, and normalization with arithmetic means highlighted in color.

Figure 2 shows all pretreated Raman spectra (grey) and the respective mean spectrum highlighted in color. The literature on the assignment of signals to the respective biochemical constituents can be obtained, for example, from [11,14]. Two slightly varying bands are clearly visible in all microorganisms containing carotenoids (*Cin*, *Kro*, *Mlu*, *Sau*, *Xde*) and their maxima range from 1132 to 1157 cm^−1^ and 1513 to 1528 cm^−1^, depending on the species.

**Figure 3:**
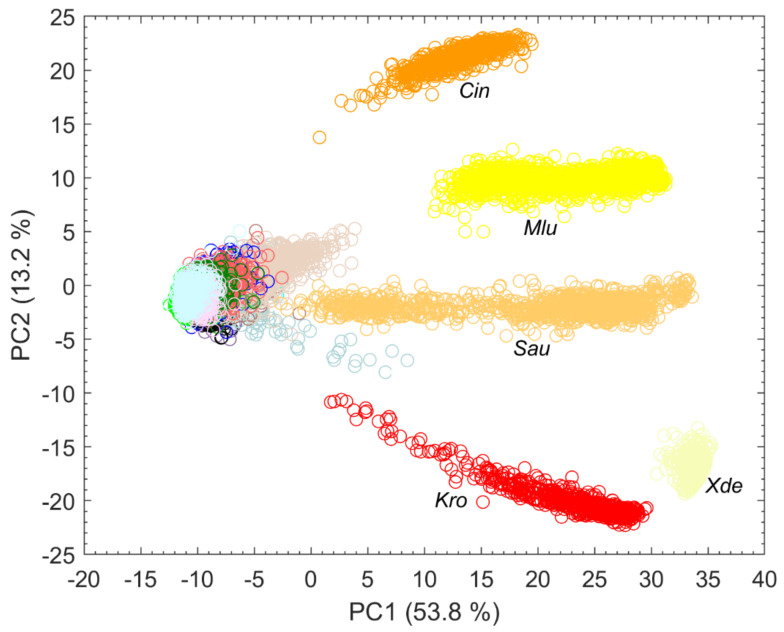
First two PCs of a PCA with explained variance in percent calculated with all spectra collected from microorganisms on the silver mirror slide (*n* = 17,651) and species abbreviation below particularly strongly separated clusters.

Splitting data into its most variance via PCA does not always mean obtaining the best information for classification [15]. It is quite possible that PCs describing less variance are better for classification as PCs describing more variance. However, looking at the first PCs can often reveal initial patterns [16]. When considering only the first two PCs, results suggest that carotenoid-containing microorganisms explain a large amount of variance in the data and can be visibly differentiated (Figure 3). Carotenoids thus seem to be a good distinguishing criterion due to their high diversity. This is also confirmed by Kumar et al. in their publication of 2015 [17]. Kumar et al. also reported about the process of photodegradation of carotenoids in various bacteria by UVA radiation via resonance Raman spectroscopy [17]. Visible light, like the employed 633 nm laser, can also lead to photodegradation of carotenoids, which can be seen, for example, in the gray background spectra of *Sau* at about 1158 and 1523 cm^−1^ in Figure 2. The carotenoids of *S. aureus* (*Sau*) and *K. rosea* (*Kro*) degraded more quickly than those contained in *X. dendrorhous* (*Xde*) or *M. luteus* (*Mlu*) (data not shown). The more scattered the data of pigmented microorganisms in Figure 3, the larger the effect of possible photodegradation seems to be, although these possible correlations need to be investigated in more detail as other influencing factors such as fluorescence degradation cannot be completely excluded.

**Figure 4:**
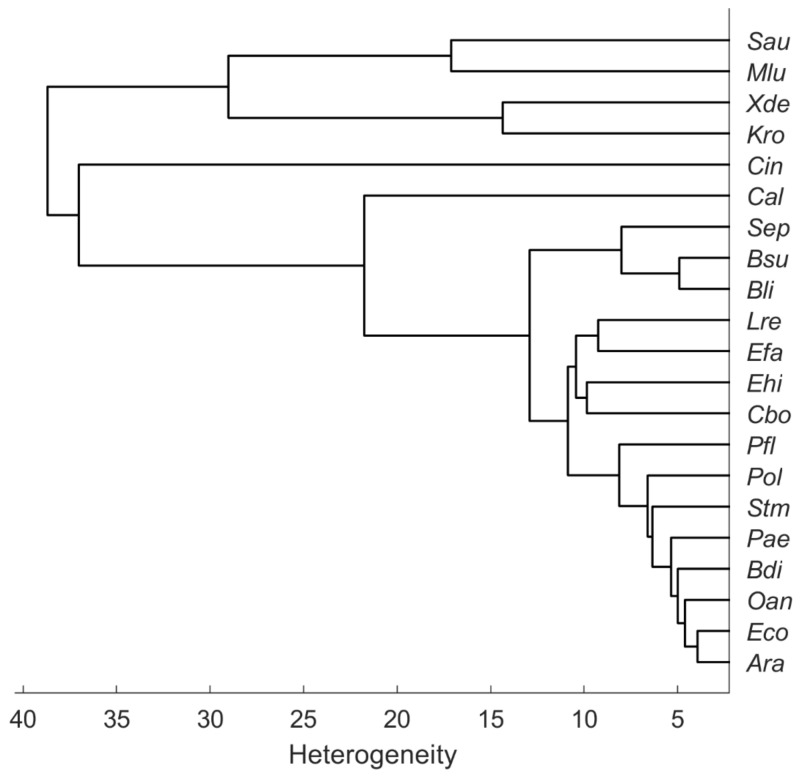
Hierarchical cluster analysis of the mean spectra recorded from microorganisms on the silver mirror slide (average linkage clustering, Euclidean distance).

The dendrogram in Figure 4 illustrates the heterogeneity or similarity of the mean spectra. The hierarchical cluster analysis (HCA) was calculated in MATLAB R2021b with average linkage clustering and the Euclidean distance. As the first two PCs of the PCA (Figure 3) suggest, the carotenoid-containing microorganisms form a separate cluster in the HCA but are relatively distinct among themselves. In their 2012 publication, Stöckel et al. showed an apparent correspondence between relationships established by Raman spectroscopy and phylogenetic or taxonomic relationships of *Bacillus* species [18]. Predictions of genetic relatedness are also possible via Raman spectroscopy and machine learning [19]. However, the phylogenetic relationship of some species may not always match Raman spectroscopic similarity. For example, the two fungal species *C. boidinii* (*Cbo*) and *C. albicans* (*Cal*) should have more similarity to each other than *C. boidinii* (*Cbo*) to the procaryote *E. hirae* (*Ehi*). Figure 4, however, shows the exact opposite. Moreover, substances that are particularly Raman active but contribute little to the phylogenetic relatedness of particular organisms could lead to phylogenetic misinterpretation in unsupervised learning procedures such as HCA; for example, there are also *S. aureus* strains without pigments [20], which means that they do not show any of the corresponding typical Raman signatures.

### 3.3. Model Development

**Figure 5:**
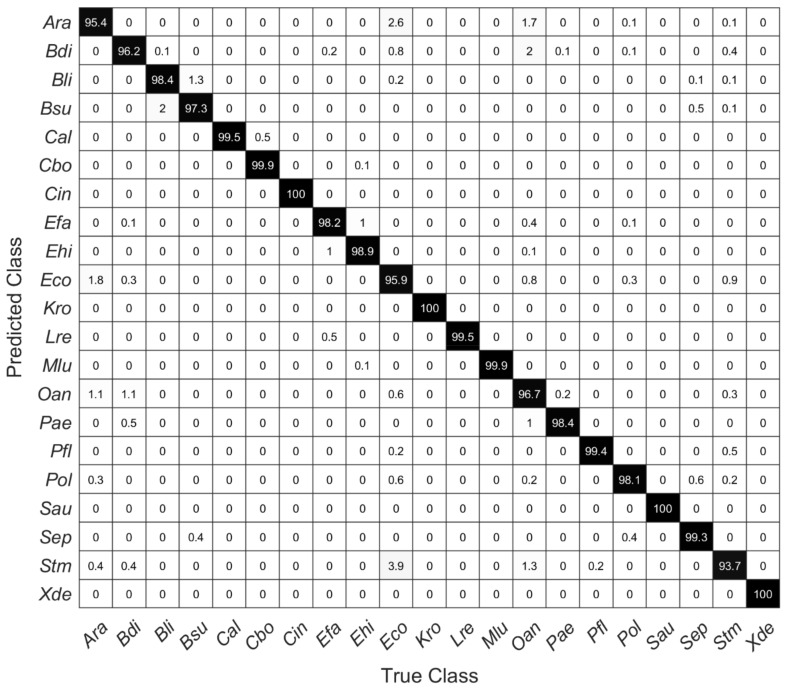
Confusion matrix of a five-fold cross-validated SVM model using the first 20 PCs showing the percentage of right and false-positive predictions of each species.

SVM models were calculated with the first 10 to 20 PCs. Table 2 shows the overall estimated prediction accuracies, which were determined via a five-fold cross-validation. Using the first 10 principal components, the overall prediction accuracy is 94.3%. Here, *Ara* was most frequently misinterpreted as *Eco* and vice versa. Similarly, using only 10 PCs, *Oan* is predicted correctly only in 82.7% (data not shown). The estimated accuracies are highest for microorganisms containing carotenoid pigments, but two of the yeasts included in this study (*Cal* and *Cbo*) can also be very well differentiated (Figure 5).

Figure 5 shows a confusion matrix of the cross-validated SVM model with 20 PCs, showing the percentage of correct and false-positive predictions of each species. Within these data, for all species except *Stm* prediction accuracy is higher than 95%. The high Raman spectroscopical similarity of *Ara* and *Eco*, which was suggested by the HCA as shown in Figure 4, is reflected in correspondingly incorrect predictions of these species. Considering all estimated accuracies, one can assume a very accurate prediction performance (98.4%) without particularly large deviations within individual included species.

### 3.4. Predictions for Independent Raman-Spectra of Microorganisms on Stainless Steel Slides

The cross-validation (Figure 5) is not suitable for estimating the predictive quality of new unknown data, e.g., in terms of unknown species of different surfaces; for this reason, a partial validation (14 out of 21 species) of the models was performed with new data. The new data were recorded from samples placed on a stainless-steel slide, but samples and data were otherwise treated completely the same as in the predictive models. Table 3 shows the prediction accuracies of all SVM models for the new data. It can be clearly seen that the overall prediction accuracy decreases compared to the cross-validated data from Table 2. Interestingly, the accuracy of the model with the first 10 PCs (72.8%) gives almost as accurate predictions as the model with 18 PCs (72.9%). Using the first 20 PCs, there is a significant rise from over 6% to a total of 80.1% accuracy.

**Figure 6:**
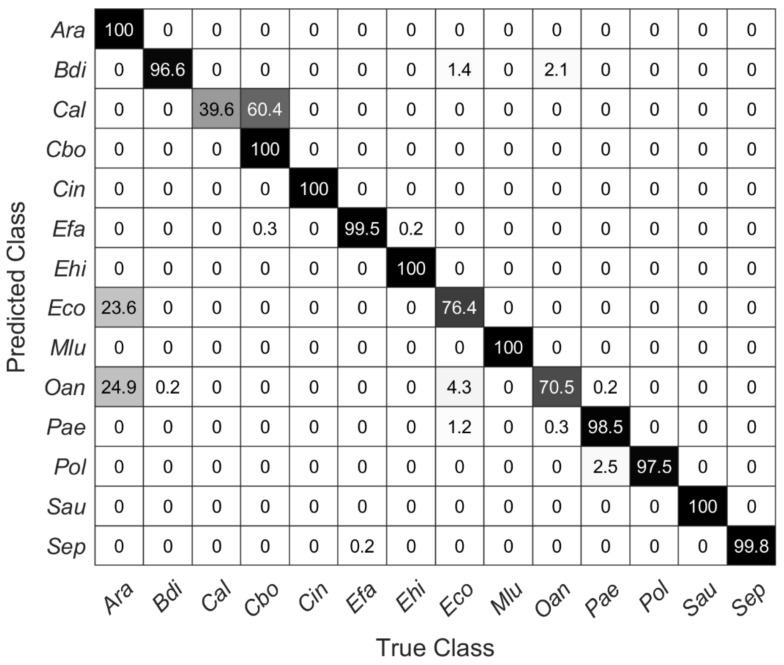
Confusion matrix showing the percentage of true and false predictions for Raman spectra of each species recorded on stainless-steel slides by using the SVM model with the first 20 PCs of data collected on silver mirror slide (585 ± 40 spectra per species).

Figure 6 shows a confusion matrix describing the true and false predictions in percent for new data recorded on the stainless-steel slide by using the SVM model with 20 PCs from above. The number of validation spectra per species is 585 ± 40. All carotenoid-containing microorganisms included here (i.e., *Cin*, *Mlu*, *Sau*) were 100% correctly predicted. Moreover, the species *Ara*, *Cbo*, and *Ehi* were predicted completely correctly. Considering the dendrogram in Figure 4, this seems reasonable for *Ehi* and *Cbo*, as these mean spectra separate quite clearly, *Ara* and *Eco*, on the other hand, show high similarities, yet *Ara* was predicted 100% correctly. Conversely, however, 23.6% of the Raman spectra of *Eco* were also predicted to be *Ara*. Likewise, *Oan* was misinterpreted as *Ara* and thus was not predicted very accurately with only 70%. With only 39.6%, *Cal* is predicted worst. There, the false-positive predictions were within the same genus (*Candida*).

**Figure 7:**
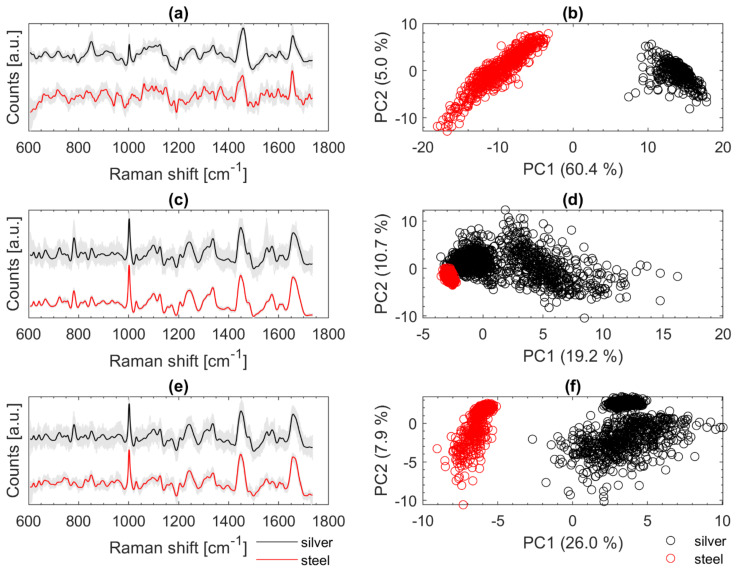
Baseline corrected and smoothed Raman spectra of *Cal* (**a**), *Eco* (**c**), and *Oan* (**e**) recorded on silver mirror slide (black) and stainless-steel slide (red); PCA calculated with the Raman spectra recorded on silver mirror slide (black) and stainless steel slide (red) of *Cal* (**b**), *Eco* (**d**), and *Oan* (**f**); for better visualization of the spectra on the left an offset was calculated in (**a**,**c**,**e**) but the spectra for calculating the PCAs on the right (**b**,**d**,**f**) were all treated the same.

Figure 7 illustrates on the left the fully treated Raman spectra in grey in the background and the highlighted mean spectra of *Cal* (a), *Eco* (c), and *Oan* (e). The mean spectra (recorded on silver mirror slide) are shown in black at the top and the spectra recorded from samples on the stainless-steel slide are shown in red at the bottom. On the right-hand side of Figure 7, the first two PCs of PCAs are shown (b, d, f) with the corresponding spectra from the left (a, c, e). The spectra of *Cal* from Figure 7a show a strong negative slope at the beginning (about 600 to 650 cm^−1^). This typically occurs when the LPF is applied to spectra with strong fluorescence background. An optimized baseline correction could minimize such effects. Moreover, the peak at about 1003 cm^−1^ (phenylalanine) is only seen in the spectra recorded on the silver slide and is not present in the other spectra (stainless steel slide). The PCA on the right (Figure 7b) illustrates the large variance within the two data sets from *Cal*. The reasons for the poorer signals of *Cal* on stainless steel are most likely not due to the measurement subsurface. Since the calibration set includes only one individual culture of *Cal*, such variations in fluorescence are not covered. Most likely the measurement conditions were more favorable (thinner sample layer, slightly younger colony material transferred with fewer fluorophores) when the data were acquired from the silver mirror slide. An expanded data set with more variation may lead to improvement. The PCA of the spectra of *Eco* (Figure 7d) shows that the spectra, which were recorded on stainless-steel slides, accumulate at the outermost edge of the calibration spectra. The variation within the validation data is very small. Spectral differences can be seen especially at 1550 cm^−1^. There, some of the reference spectra (silver slide) show an additional signature. For *Oan* (Figure 7e), a slight effect due to the LPF can also be observed as for *Cal* (Figure 7a). A striking feature of the spectra of *Oan* is the strongly pronounced signature at 780 cm^−1^ from the spectra recorded on the silver slide. Signals in this region are mainly triggered by DNA components (phosphate bond, cytosine, uracil, thymine) [11,14,21]. This band is only very weakly pronounced in the *Oan* spectra on stainless steel (Figure 7e).

## 4. Conclusions

Both materials, silver mirror slide and polished stainless-steel slide, are proven to be suitable for Raman microscopic analysis of microorganisms with our measurement setup and the signal yield is generally much higher than with glass slides (Figure 1). The partially stronger fluorescence disturbance of a few samples on the stainless-steel substrate is most likely not due to the substrate but to the samples. The tests show that the developed SVM model with 20 PCs performs very effectively in the cross-validation (98.7%). Predictions for completely new and independent data, which were also collected on a different substrate, are also very accurate for most species. For 3 out of 14 validated species, the prediction quality is below 95% and the overall average is around 80%. By augmenting the data set collected on the silver mirror slide with the validation data (collected on a stainless-steel slide), increasing the size of the data set with more independent samples, and optimizing data pretreatment, the models are expected to improve in accuracy. Advances in artificial intelligence and access to related software also offer new opportunities we pursue.

## Figures and Tables

**Table 1:** List of species included in the database with their DSM-No. and additional information on the number of spectra, number of independently grown cultures, used exposure time, accumulations per spectrum, and growth conditions; all spectra have been recorded with an excitation wavelength of 633 nm at about 3.5 mW laser power on sample.

Microorganism	Abbreviation	DSM-No.	Spectra	Independent Cultures	Exposure Time (Seconds); Accumulations	Nutrition Media	Cultivation Time
*Acinetobacter radioresistens*	*Ara*	6976	725	1	1.5; 20	TSA	24 h
*Brevundimonas diminuta*	*Bdi*	7234	897	3			
*Bacillus licheniformis*	*Bli*	13	1240	2			
*Bacillus subtilis*	*Bsu*	10	790	1			
*Candida albicans*	*Cal*	1386	400	1		MEA	
*Candida boidinii*	*Cbo*	70,034	686	1			
*Chryseobacterium indolgenes*	*Cin*	16,777	684	1	1.5; 15	TSA	
*Enterococcus faecium*	*Efa*	2146	680	2	1.5; 20		
*Enterococcus hirae*	*Ehi*	3320	1423	2			
*Escherichia coli*	*Eco*	423	1190	3			
*Kocuria rosea*	*Kro*	own isolate	639	1	1.5; 15		
*Lactobacillus reuteri*	*Lre*	20015	420	1	1.5; 20		48 h
*Micrococcus luteus*	*Mlu*	1790	1842	6	1.5; 15		24 h
*Ochrobactrum anthropi*	*Oan*	6882	1045	2	1.5; 20		
*Pseudomonas aeruginosa*	*Pae*	939	385	1			
*Pseudomonas fluorescens*	*Pfl*	50,090	654	1			
*Pseudomonas oleovorans* subsp *lubricantis*	*Pol*	21,016	632	1			
*Staphylococcus aureus*	*Sau*	799	1094	3	1.5; 15		
*Staphylococcus epidermidis*	*Sep*	1798	1111	2	1.5; 20		
*Stenotrophomonas maltophilia*	*Stm*	50,170	460	1	1.5; 20		
*Xanthophyllomyces dendrorhous*	*Xde*	5626	658	1	1.5; 15	MEA	

**Table 2:** Number of used PCs for SVM models and estimated prediction accuracy by five-fold cross-validation.

**Number of used PCs for SVM**	10	11	12	13	14	15	16	17	18	19	20
**Estimated prediction accuracy in %**	94.3	94.6	95	95.6	96.1	97.2	97.4	97.8	98	98.2	98.4

**Table 3:** Number of used PCs for SVM models and validated prediction accuracy for new data collected from samples on stainless-steel slide.

**Number of used PCs for SVM**	10	11	12	13	14	15	16	17	18	19	20
**Validated prediction accuracy in %**	72.8	64.6	62.3	70.7	70	72.8	73.9	72.2	72.9	73.8	80.1
